# Mean platelet volume and platelet distribution width serve as prognostic biomarkers in skull base chordoma: a retrospective study

**DOI:** 10.1186/s12885-020-07497-7

**Published:** 2020-10-12

**Authors:** Mingxuan Li, Jiwei Bai, Shuai Wang, Yixuan Zhai, Shuheng Zhang, Chuzhong Li, Jiang Du, Yazhuo Zhang

**Affiliations:** 1 Beijing Neurosurgical Institute, Capital Medical University, No.119 South Fourth Ring West Road, Fengtai District, Beijing, 100070 China; 2 Department of Neurosurgery, Beijing Tiantan Hospital, Capital Medical University, Beijing, China; 3 Department of Neurosurgery, The First Affiliated Hospital of Zhengzhou University, Zhengzhou, China; 4 Department of Neurosurgery, Anshan Central Hospital, Anshan, China; 5 Department of Neuropathology, Beijing Neurosurgical Institute, Capital Medical University, Beijing, China; 6 Beijing Institute for Brain Disorders Brain Tumor Center, Beijing, China; 7 China National Clinical Research Center for Neurological Diseases, Beijing, China; 8 Key Laboratory of Central Nervous System Injury Research, Capital Medical University, Beijing, China

**Keywords:** Skull base chordoma, Platelet, Mean platelet volume, Platelet distribution width, Prognostic marker

## Abstract

**Background:**

Increasing studies have demonstrated that activated platelets play an essential role in tumour progression. However, the level and prognostic role of platelet indices in chordoma patients remain unclear. The aim of the current study was to characterize the prognostic performance of platelet count (PLT), mean platelet volume (MPV) and platelet distribution width (PDW) in skull base chordoma patients.

**Methods:**

187 primary skull base chordoma patients between January 2008 and September 2014 were enrolled in this retrospective study. The optimal cut-off values were determined by X-tile software, and the correlations between PLT, MPV, PDW and clinicopathological features were further analysed. Kaplan-Meier curve and Cox regression analysis were used for survival analysis.

**Results:**

The values of preoperative PTL, MPV and PDW ranged from 104 to 501 × 10^9^/L, 6.7 to 14.2 fl, and 7.8 to 26.2%, respectively. Elevated PLT was associated with larger tumour volume (*p* = 0.002). Kaplan-Meier survival analysis revealed that increased MPV and PDW were associated with shorter overall survival (*p* = 0.022 and 0.008, respectively). Importantly, multivariate Cox analysis demonstrated that elevated PDW was an independent unfavourable predictive factor for overall survival (hazard ratio (HR), 2.154, 95% confidence interval (CI), 1.258–3.688, *p* = 0.005).

**Conclusions:**

Our data show that elevated MPV and PDW are associated with poor outcomes in skull base chordoma and that PDW may be helpful to identify patients with high risk.

## Background

Skull base chordoma is a slow-growing cancer of the bone system originating from notochord remnants, with a morbidity of less than 1 per million and a slight preference of male patients [1, 2]. The current first choice of treatment for skull base chordoma patients remains complete surgical resection with recommended postoperative proton-beam therapy [3, 4]. However, the therapy of skull base chordoma patients is still challenging owing to the difficulty of radical resection, subsequent local recurrence, resistance to classical chemoradiotherapy and limited value of targeted therapy [1, 5]. The identification of effective prognostic markers for potential risk stratification and to better select individual treatment strategies are urgently needed to prolong the life span and reduce the financial burden of skull base chordoma patients.

Increasing evidence indicates that platelets derived from megakaryocytes play an essential role in the tumour initiation, development and metastasis through several aspects such as tumour cell growth and invasion, abnormal angiogenesis, and inflammatory process [6, 7]. Moreover, activated platelets are closely correlated with cancer-associated thrombosis via interactions with tumour cells, neutrophils and monocytes. Recent studies revealed that increased platelet count (PLT) was observed in various cancers and it was closely associated with poor outcomes in colorectal cancer [8], non-small cell lung cancer [9], glioblastoma [10] and epithelial ovarian carcinoma [11], indicating the potential role of anti-platelet therapy in comprehensive cancer therapy.

Mean platelet volume (MPV), an index characterizing the size of platelets, is a valuable indicator of platelet activation and changes in platelet production [12, 13]. In addition, preoperative MPV was found to be elevated in various cancer patients compared to that in healthy people, and it has been recognized as a useful diagnostic marker in various diseases, including malignancies, cardiovascular disease and stroke [12]. Moreover, further studies indicate that MPV can act as an effective prognostic indicator for outcome in patients with cancers such as esophageal squamous cell cancer and colorectal cancer [14]. Platelet distribution width (PDW), another platelet associated indicator evaluating the coefficient of variation in platelet dimension, is considered a hallmark of platelet morphology and is widely used for the differential diagnosis of thrombocytopenia [15]. Besides, an increasing numbers of studies have revealed that PDW is elevated in cancer patients and can independently predict patient survival in various malignancies [16, 17].

However, to our knowledge, few studies have evaluated the preoperative levels and prognostic roles of these platelet-associated indexes in skull base chordoma until now. Thus, the current study aimed to characterize the preoperative levels of PLT, MPV and PDW and explore their correlations in primary skull base chordoma patients. We also assessed the relationships of PLT, MPV, and PDW with clinical factors and patient outcome in skull base chordoma.

## Methods

### Study population and data collection

This retrospective study analysed skull base chordoma patients who received operations at Beijing Tiantan Hospital from January 2008 to September 2014. Patients with histopathologically confirmed skull base chordoma and no history of preoperative radiotherapy or chemotherapy were included. Patients with any of the following conditions were excluded: (1) fuzzy pathological diagnosis; (2) incomplete clinical data and/or preoperative laboratory tests; (3) history of preoperative treatment (operation, chemoradiotherapy); (4) evidence of other malignancies, infection, inflammation or autoimmune disease, haematological disease or blood transfusion; and (5) unavailable follow up information. Accordingly, 187 primary skull base chordoma patients were included in the study. The ethical committee of Beijing Tiantan Hospital approved the current study and informed consent was received from the enrolled patients.

The clinicopathological data of each patient including age at diagnosis, patient sex, symptoms, pathological type, tumour size, tumour texture and blood supply, brainstem involvement, posterior cranial nerve involvement and preoperative laboratory tests containing PLT (10^9^/L), MPV (fl), and PDW (%) were acquired from medical records. The extent of resection was assessed as total resection or non-total resection according to the pre- and postoperative image examinations [18].

### Patients’ treatment and follow up

All patients were treated with surgical resection (endoscopic endonasal approach for 73 patients, endoscopic transoral approach for 6 patients, transcranial approach for 108 patients), and no patients received exclusive radiotherapy alone. For patients with a non-total resection, postoperative adjuvant radiotherapy was recommended.

Survival data were acquired from each patient via regular follow up, and the last time of follow up was October 2019. Patients were periodically followed up at the interval of 3 to 6 months for the first 2 years after the operation, and then annually. Clinical examinations and contrast-enhanced MRI were routinely used at each follow-up time. Overall survival (OS), calculated as the time between the date of tumour resection to the date of death or the last follow-up, was used for survival analysis. The mean follow up time was 72.41 months (range, 3–141 months; median, 74 months).

### Definition of cutoff values for PLT, MPV and PDW

X-tile 3.6.1 software (Yale University, New Haven, CT, USA) was used to find the optimal cutoff values of each index for OS analysis [19]. In brief, the patients were divided into two groups according to certain values, and a subsequent log-rank test comparing the two groups was performed. The value with the minimum *p* value was defined as the best cutoff value.

### Statistical analysis

All statistical analyses were conducted by SPSS 19.0 software (IBM, Armonk, NY, USA) and GraphPad Prism (Version 7.0, GraphPad, La Jolla, CA, USA) was used for graph construction. Continuous variables were listed as the median or mean ± standard deviation, and categorical variables were expressed as the frequency. The chi-square test was used for comparisons between categorical variables. Correlations between PLT MPV and PDW were analysed using Pearson correlation. The Kaplan-Meier method and subsequent log-rank test were used for OS analysis between groups. Variables with a *p* value < 0.05 in univariate Cox analysis were enrolled in multivariate Cox analysis to evaluate independence. Statistical significance was considered if the *p* value was less than 0.05 in 2-sided tests.

## Results

### Summary of patients

**Fig. 1:**
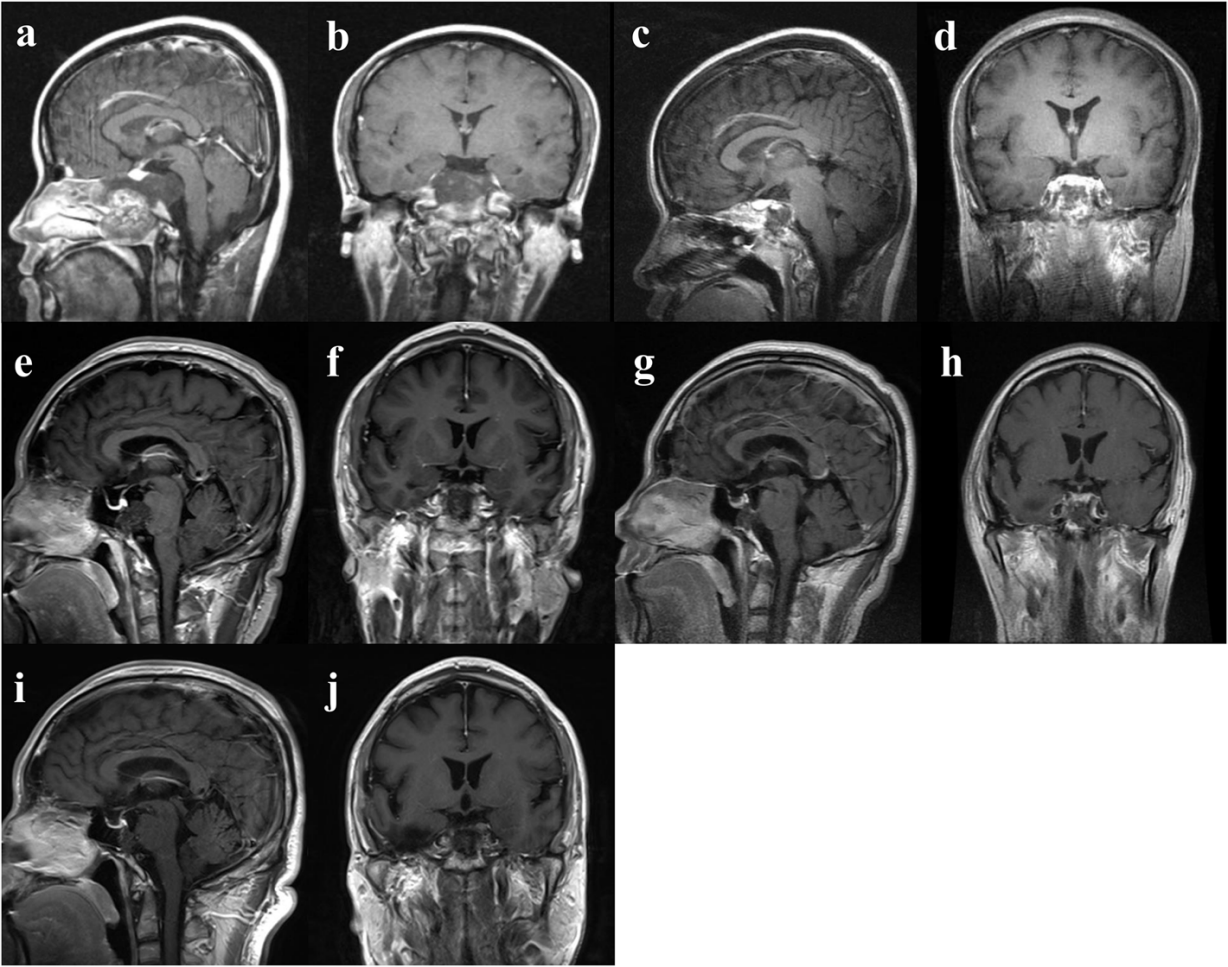
Representative preoperative and postoperative MR images showing the definition of total and non-total resection, as well as the value of combined treatment. **a-d** preoperative and postoperative images of one case who received total resection and the patient got no recurrence during the follow up. **e-h** preoperative and postoperative images of another case who received non-total resection and following adjuvant radiotherapy. **i-j** After surgery and postoperative adjuvant radiotherapy, the case shown in e-h had no sign of recurrence on the 70 months follow up images

Patient descriptive characteristics are reported in Table 1. A total of 187 skull base chordoma patients meeting the inclusion criteria were enrolled in this retrospective study, including 98 males and 89 females with a mean (± SD) age at diagnosis of 40.1 (± 15.3) years old. Tumour volumes varied from 1740.5 to 258,024.6 mm^3^ (mean ± SD, 31729.8 ± 33,238.5). The most common symptoms of skull base chordoma patients were headache (88 patients), diplopia (68 patients), and blurred vision (61 patients). Fifty-seven patients had soft tumours and the other 130 patients had hard/moderate tumor. A total of 109 patients had a rich tumor blood supply, and 78 patients with poor/moderate tumour blood supply. The numbers of patients with classical, chondroid and dedifferentiated chordoma were 126, 61, and 0, respectively. A total of 118 patients had brainstem involvement, and 69 patients had posterior cranial nerve involvement. Regarding surgical outcome, 41 patients received total resection and the remaining 146 patients received non-total resection (Fig. 1). 72 patients received postoperative radiotherapy. Among them, 42 (58.3%) patients received the gamma knife; 8 (11%) patients received proton beam therapy; 6 (8.3%) patients received other forms of radiotherapy (1 carbon ion therapy, 1 cyberknife, and 4 intensity modulated radiotherapy); and the detailed forms of radiotherapy were unknown in 16 (22.2%) patients (Table 1).

**Table 1:** Summary of 187 skull base chordoma patients

Variable	Number of patients
Age, years, mean ± SD	40.1 ± 15.3
Sex	
Male	98
Female	89
Tumour volume, mm^3^, mean ± SD (range)	31,729.8 ± 33,238.5 (1740.4–258,024.6)
Most Common symptoms	
Headache	88
Diplopia	68
Blurred vision	61
Vision field defect	43
Dizziness	30
Tumour texture	
Soft	57
Hard/moderate	130
Tumour blood supply	
Rich	109
Poor/moderate	78
Pathology type	
Classical	126
Chondroid	61
Dedifferentiated	0
Brainstem involvement	
No	69
Yes	118
Posterior cranial nerve involvement	
No	118
Yes	69
Surgical approach	
Endoscopic endonasal	73
Endoscopic transoral	6
Transcranial	108
Degree of resection	
Total resection	41
Non-total resection	146
Postoperative radiotherapy	
No	115
Yes	72
Gamma knife	42
Proton beam therapy	8
Carbon ion therapy	1
Cyberknife	1
Intensity modulated radiotherapy	4
Unknown	16
Median PLT, 10^9^/L (range)	234 (104–501)
Median MPV, fl (range)	10.2 (6.7–14.2)
Median PDW, % (range)	11.8 (7.8–26.2)
Death during follow up	72

*SD* standard deviation, *PLT* platelet count, *MPV* mean platelet volume, *PDW* platelet distribution width

### PLT, MPV, and PDW levels in skull base chordoma patients

The median (range) levels of PTL, MPV and PDW were 234 (104–501) × 10^9^/L, 10.2 (6.7–14.2) fl, and 11.8 (7.8–26.2) %, respectively (Table 1). We then used X-tile software to find the optimal cutoff value of each index, and the cutoff values of PLT, MPV and PDW were 266, 11.9 and 14.5, respectively (see Additional file 1). Accordingly, the patients were divided into two groups for further analysis: 142 (75.9%) patients with PLT ≥266 and 45 (24.1%) patients with PLT < 266; 165 (88.2%) patients in the MPV ≥11.9 group and 22 (11.8%) patients in the MPV < 11.9 group; and 156 (83.4%) patients with PDW ≥14.5 and 31 (16.6%) patients with PDW < 14.5.

### Relationships between PLT, MPV, and PDW and clinical variables

We also analysed the correlations between PLT, MPV, and PDW and clinical parameters. As shown in Table 2, only higher PLT (PLT ≥266) was associated with larger tumour volume (*p* = 0.002). No significant differences were found between PLT, MPV, and PDW and clinicopathological features including patient sex, age at diagnosis, pathological types, tumour texture, tumour blood supply, brainstem involvement and posterior cranial nerve involvement. Of note, patients with high PLT tended to have tumours with rich blood supply (*p* = 0.098). Additionally, a larger tumour volume seemed to be more common in patients with PDW ≥14.5 (*p* = 0.108).

**Table 2:** Relationship between PLT, MPV, PDW and clinicopathological features in skull base chordoma

Variables	PLT (10^9^/L), N			MPV (fl), N			PDW (%), N		
	< 266	≥266	*P* value	< 11.9	≥11.9	*P* value	< 14.5	≥14.5	*P* value
Sex			0.376			0.487			0.377
Male	77	21		88	10		84	14	
Female	65	24		77	12		72	17	
Age			0.187			0.262			0.203
≤ 55	114	40		134	20		126	28	
> 55	28	5		31	2		30	3	
Tumour volume			0.002*			0.439			0.108
≤ 20,000 mm^3^	78	13		82	9		80	11	
> 20,000 mm^3^	64	32		83	13		76	20	
Texture			0.313			0.400			0.536
Soft	46	11		52	5		49	8	
Hard/moderate	96	34		113	17		107	23	
Blood supply			0.098			0.705			0.978
Rich	78	31		97	12		91	18	
Poor/ moderate	64	14		68	10		65	13	
Pathology type			0.907			0.569			0.376
Classical	96	30		110	16		103	23	
Chondroid	46	15		55	6		53	8	
Brainstem involvement			0.356			0.678			0.858
No	55	14		60	9		58	11	
Yes	87	31		105	13		98	20	
Posterior cranial nerve involvement			0.229			0.678			0.858
No	93	25		105	13		98	20	
Yes	49	20		60	9		58	11	
Total	142	45		165	22		156	31	

* indicate *p* < 0.05

*PLT* platelet count, *MPV* mean platelet volume, *PDW* platelet distribution width;

We then analysed the potential correlation among PLT, MPV and PDW. Our results indicated that PDW was negatively correlated with PLT (r = − 0.344, *p* < 0.001), however, a strong positive correlation was observed between PDW and MPV (r = 0.844, p < 0.001).

### Analysis of the association of PLT, MPV and PDW with patient outcomes

**Fig. 2:**
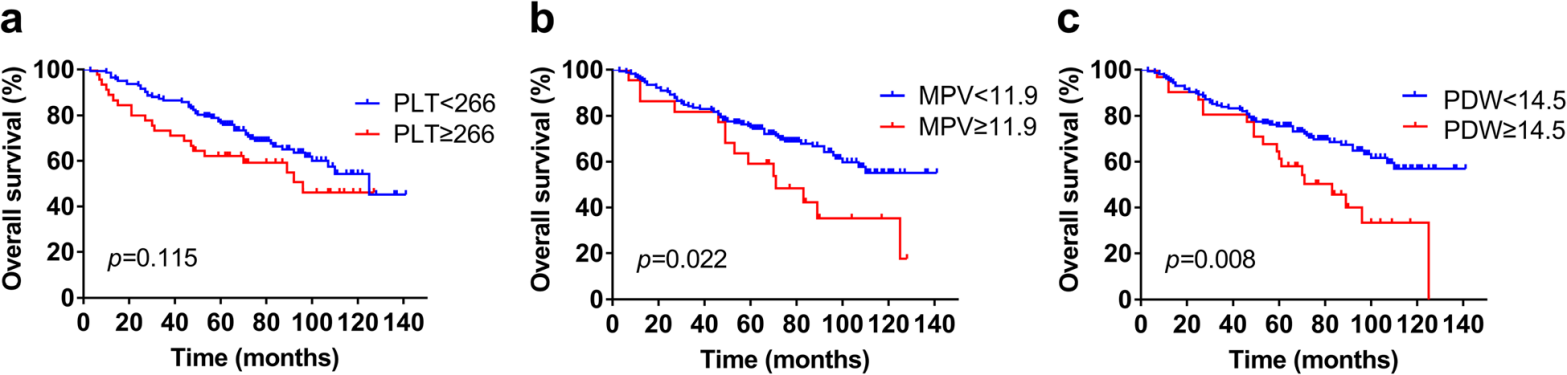
Kaplan-Meier curves of PLT, MPV and PDW in skull base chordoma. **a** PLT and OS. **b** MPV and OS. **c** PDW and OS. PLT, platelet count; MPV, mean platelet volume; PDW, platelet distribution width; OS, overall survival

A total of 72 (38.5%) patients died during the follow-up, and the 5-year OS rate was 68.4% in the current study. Kaplan-Meier analysis demonstrated a shorter OS time (mean OS time, 82.3 months, 5-year OS rate, 62.1%) in the PLT ≥266 group than that in the PLT < 266 group (mean OS time, 102.7 months; 5-year OS rate, 75.8%), though the *p* value was 0.115 (Fig. 2a). For MPV, patients with MPV ≥11.9 had a worse OS (mean OS time, 77.7 months; 5-year OS rate, 59.1%) than patients with MPV < 11.9 (mean OS time, 103.0 months; 5-year OS rate, 74.36%) (*p* = 0.022, Fig. 2b). Moreover, the OS time of patients with PDW ≥14.5 (mean OS time, 78.2 months; 5-year OS rate 58.1%) was significantly shorter than that of patients with PDW < 14.5 (mean OS time, 104.2 months; 5-year OS rate 75.6%) (*p* = 0.008, Fig. 2c).

**Fig. 3:**
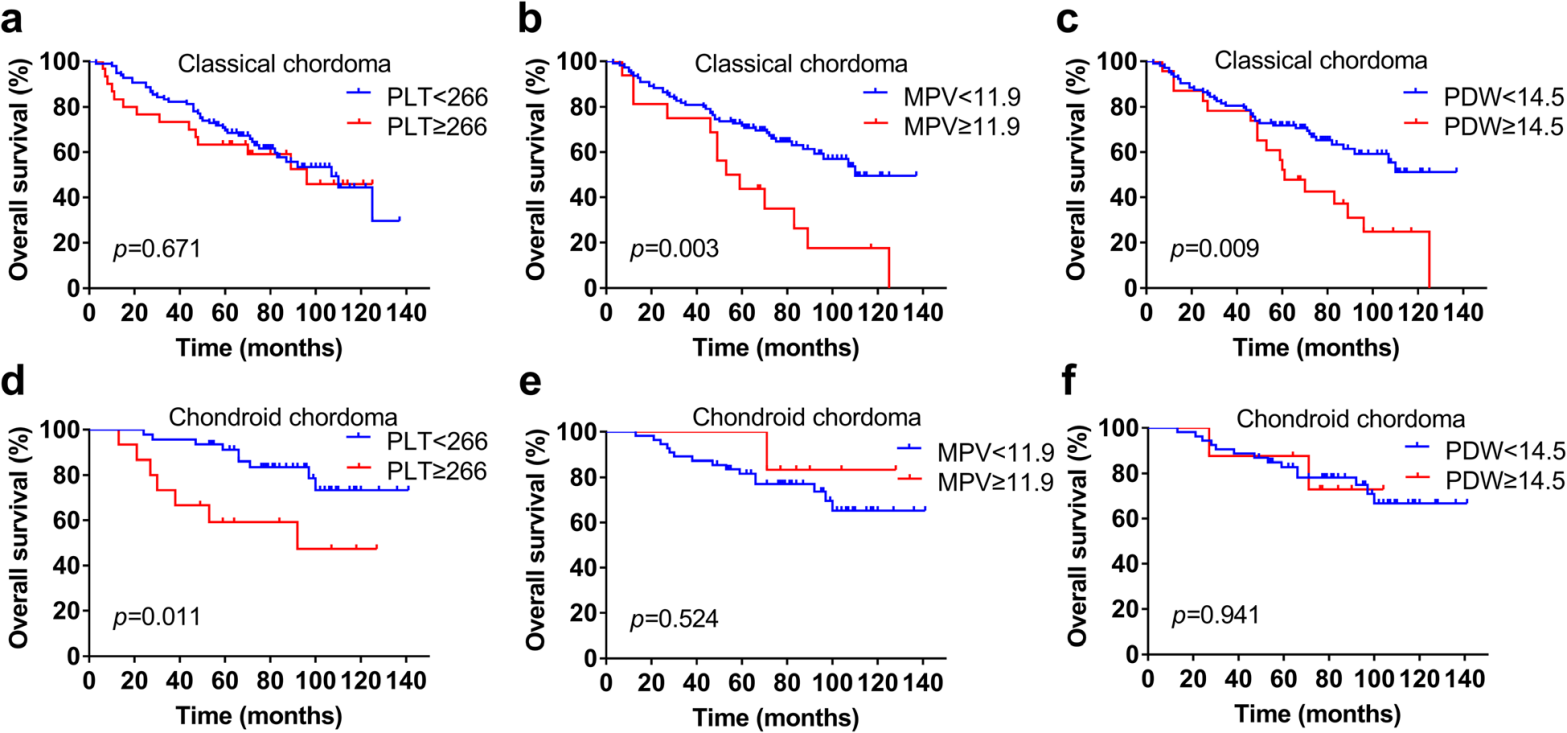
Kaplan-Meier curves of PLT, MPV and PDW in different pathological types of skull base chordoma. **a** OS analysis of PLT in classical chordoma patients. **b** OS analysis of MPV in classical chordoma patients. **c** OS analysis of PDW in classical chordoma patients. **d** OS analysis of PLT in chondroid chordoma patients. **e** OS analysis of MPV in chondroid chordoma patients. **f** OS analysis of PDW in chondroid chordoma patients. PLT, platelet count; MPV, mean platelet volume; PDW, platelet distribution width; OS, overall survival

**Fig. 4:**
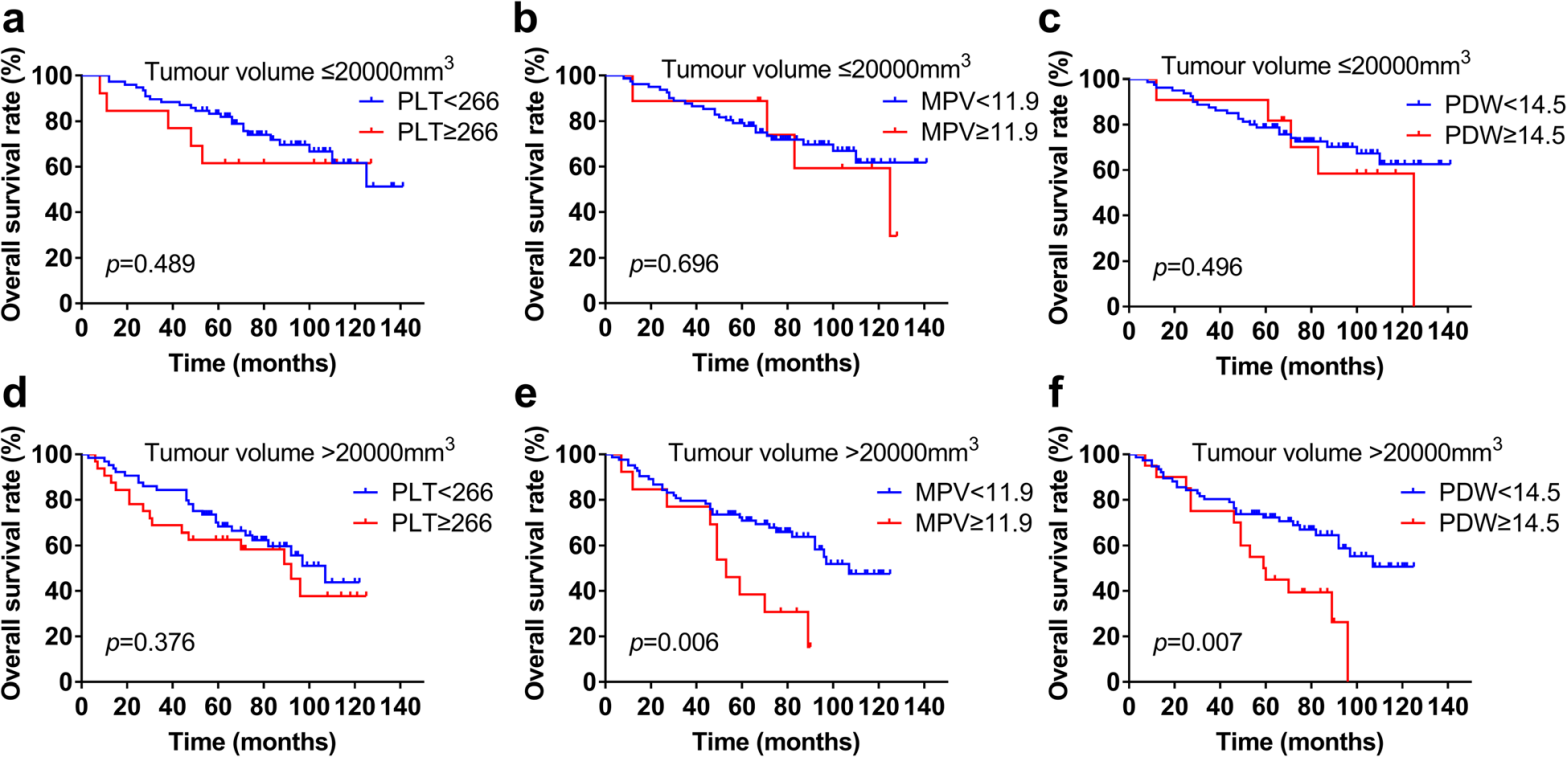
Kaplan-Meier curves of PLT, MPV and PDW in skull base chordoma with different tumor volumes. **a** OS analysis of PLT in tumor volume ≤ 20,000 mm^3^ patients. **b** OS analysis of MPV in tumor volume ≤ 20,000 mm^3^ patients. **c** OS analysis of PDW in tumor volume ≤ 20,000 mm^3^ patients. **d** OS analysis of PLT in tumor volume > 20,000 mm^3^ patients. **e** OS analysis of MPV in tumor volume > 20,000 mm^3^ patients. **f** OS analysis of PDW in tumor volume > 20,000 mm^3^ patients. PLT, platelet count; MPV, mean platelet volume; PDW, platelet distribution width; OS, overall survival

Further subgroup analysis of different tumour pathologies showed that higher PLT (*p* = 0.671, Fig. 3a) showed no prognostic value in classical chordoma patients, while higher MPV (*p* = 0.003, Fig. 3b) and PDW (*p* = 0.009, Fig. 3c) were associated with poor OS in the classical chordoma subgroup. Conversely, for chondroid chordoma patients, higher PLT (*p* = 0.011, Fig. 3d) rather than higher MPV (*p* = 0.524, Fig. 3e) or PDW (*p* = 0.941, Fig. 3f) was associated with a shorter OS time. In addition, for patients with different tumour volumes, the differences between the different PLT, MPV and PDW groups were not significant in tumour volume ≤ 20,000 mm^3^ patients (*p* = 0.489, *p* = 0.696, *p* = 0.496, respectively). The OS between the different PLT groups showed no significance (*p* = 0.376); however, MPV (*p* = 0.006) and PDW (*p* = 0.007) still showed prognostic value in patients with tumour volume > 20,000 mm^3^ (Fig. 4).

Univariate Cox analysis revealed that age at diagnosis (hazard ratio (HR), 1.852; 95% confidence interval (CI), 1.072–3.198; *p* = 0.027), tumour volume (HR, 1.697; 95% CI, 1.056–2.728; *p* = 0.029), tumour blood supply (HR, 0.523; 95% CI, 0.314–0.870; *p* = 0.013), tumour pathology type (HR, 0.493; 95% CI, 0.283–0.861; *p* = 0.013), degree of resection (HR, 3.390; 95% CI, 1.552–7.405; *p* = 0.002), tumour recurrence (HR, 9.549; 95% CI, 3.482–26.183; *p* < 0.001), MPV (HR, 1.957; 95% CI, 1.090–3.514; *p* = 0.025) and PDW (HR, 2.013; 95% CI, 1.191–3.405; *p* = 0.009) were associated with poor OS, while PLT showed no significance (*p* = 0.119). To identify potential independent factors, further multivariate Cox analysis including these 8 variables was carried out, and the results showed that PDW (HR, 2.154; 95% CI, 1.258–3.688; *p* = 0.005), age at diagnosis (HR, 1.791; 95% CI, 1.023–3.315; *p* = 0.042), degree of resection (HR, 2.585; 95% CI, 1.172–5.704; *p* = 0.019) and tumour recurrence (HR, 7.460; 95% CI, 2.701–20.599; *p* < 0.001) were independent indicators of OS (Table 3).

**Table 3:** Univariable and multivariable Cox analysis of OS in skull base chordoma patients

Variables	Univariable analysis			Multivariable analysis		
	HR	95% CI	*P* value	HR	95% CI	*P* value
Age (> 55 versus ≤55 years)	1.852	1.072–3.198	0.027*	1.791	1.023–3.135	0.042*
Sex (female versus male)	0.975	0.613–1.551	0.916			
Tumour volume (> 20,000 versus ≤20000mm^3^)	1.697	1.056–2.728	0.029*	NA	NA	0.569
Texture (hard/moderate versus soft)	1.612	0.935–2.780	0.086			
Blood supply (poor/moderate versus rich)	0.523	0.314–0.870	0.013*	NA	NA	0.157
Pathology (chondroid versus classical)	0.493	0.283–0.861	0.013*	NA	NA	0.128
Brainstem involvement (yes versus no)	1.013	0.630–1.629	0.956			
Posterior cranial nerve involvement (yes versus no)	1.287	0.802–2.064	0.295			
Degree of resection (non-total versus total resection)	3.390	1.552–7.405	0.002*	2.585	1.172–5.704	0.019*
Postoperative radiotherapy (yes versus no)	0.794	0.479–1.317	0.371			
Tumour recurrence (yes versus no)	9.549	3.482–26.183	< 0.001*	7.460	2.701–20.599	< 0.001*
PLT (≥266 versus < 266)	1.499	0.902–2.494	0.119			
MPV (≥11.9 versus < 11.9)	1.957	1.090–3.514	0.025*	NA	NA	0.527
PDW (≥14.5 versus < 14.5)	2.013	1.191–3.405	0.009*	2.154	1.258–3.688	0.005*

* indicate *p* < 0.05

*OS* overall survival, *HR* hazard ratio, *CI* confidence interval, *PLT* platelet count, *MPV* mean platelet volume, *PDW* platelet distribution width, *NA* not acquired

## Discussion

To our knowledge, this study was first to evaluate the prognostic role of preoperative platelet associated indexes (PLT, MPV and PDW) in skull base chordoma. Our data demonstrated that preoperative MPV and PDW rather than PLT were associated with patient OS. Multivariate Cox analysis indicated that high PDW (PDW ≥14.5) was an independent prognostic indicator of survival in skull base chordoma patients. In addition, our data confirmed that tumour recurrence and degree of resection were associated with OS [1, 20–22]. Our data revealed that MPV and PDW may be practical clinical biomarkers for prognosis in skull base chordoma due to the easy availability and relative affordability in daily clinical practice.

Platelets were identified to be involved in the process of tumour progression by numerous researches, however, the prognostic value of PLT remains disputable in different cancers, even in patients with the same kind of tumours [6, 23]. Increasing studies have indicated that an elevated preoperative PLT was associated with unfavourable prognosis in lung cancer, hepatocellular carcinoma and colorectal cancer [9, 24], however, some studies found that a lower PLT rather than a higher PLT predicted poor survival in hepatocellular carcinoma [25, 26]. This inconsistency may be explained by different cutoff values of PLT, differences in the follow-up time, potential selection bias of the study population, and tumour heterogeneity [27]. In the current study, similar to previous studies identifying PLT as a risk factor for survival, our data revealed that patients with preoperative PLT ≥266 tended to have a shorter OS time than patients with PLT < 266 (mean OS time, 82.3 months versus 102.7 months), indicating the potential relation between high PLT and poor outcome in skull base chordoma, though the *p* value between groups was > 0.05. Additional studies assessing the prognostic performance of PLT in skull base chordoma, and research exploring whether PLT is increased in skull base chordoma patients compared to healthy controls are highly warranted.

Interestingly, changes in MPV and PDW in patients with dissimilar tumours seemed controversial as well. Preoperative MPV and PDW were found to be increased and serve as risk factors for survival in various malignancies, including colorectal cancers and stomach cancers [12]. However, several researches showed that MPV was decreased in non-small-cell lung cancer patients, and subsequent survival results showed that MPV could act as a protective factor for patient outcomes [28]. In addition, a study indicated that PDW was decreased in breast cancer patients compared to controls, though patients with relatively high PDW were still associated with inferior outcomes [29]. In this study, our data indicated that preoperative MPV ≥11.9 and PDW ≥14.5 were associated with unfavourable OS in skull base chordoma patients, and PDW ≥14.5 was further identified as an effective independent prognostic indicator for OS, although MPV failed to be statistically significant in the multivariable Cox model. Further exploration of this conflict may deepen our understanding of the clinical implications and mechanisms of platelet-associated indicators in cancer patients.

The underlying mechanisms of elevated PLT, MPV and PDW levels in tumour progression remain to be elucidated. Increased PLT and platelet activation induced by the secretion of cytokines from tumour cells is associated with hypercoagulable state and thrombosis in patients, which are tightly associated with shorter survival [30]. In addition, tumour cells can escape the tumour immunity with the help of the hypercoagulable microenvironment and physical barrier by thrombosis [6]. Increased PLT can promote CD40 ligand production and contribute to the inflammatory response [31], and the inflammatory response participates in tumourigenesis and tumour development through several aspects, such as the induction of reactive oxygen species and subsequent DNA damage, promotion of tumour cell growth and angiogenesis via the secretion of various cytokines and enhanced tumour cell adhesion, and the induction of potential tumour micrometastasis [32]. In addition, platelet-derived growth factor (PDGF) family secreted by platelets plays a vital role in cell proliferation and invasion via binding to its respective receptors [33], and recent studies have revealed that PDGF receptor B is significantly expressed and associated with unfavourable outcome in skull base chordoma [34, 35]. Moreover, vascular endothelial growth factor (VEGF), which is secreted by platelet, contributes to tumour angiogenesis and serves as a predictor of tumor progression in chordoma patients receiving sorafenib [36]. MPV and PDW were considered as indicators of platelet activation [17, 37], and previous studies reported that the aberrance of MPV and PDW levels may be correlated with megakaryocyte dysfunction, heterogeneous demarcation and abnormal bone marrow haematopoietic system [38], and the release of inflammatory cytokines, including interleukin-6 and several colony stimulating factors such as granulocyte colony stimulating factors, by tumour cells can regulate megakaryocytic maturation and subsequent platelet synthesis and size [39]. As an essential proinflammatory mediator, interleukin-6 has been identified to promote oncogenesis by regulation of tumour cells survival, metabolism and angiogenesis [40]. We thus hypothesized that skull base patients with high MPV or PDW may have aberrant levels of cytokines such as interleukin-6 and abnormal inflammatory responses, leading to tumour progression and poor outcomes [32]. Interesting, several cytokines including interleukin-6 and tumor necrosis factor-alpha were reported to be elevated in chordoma patients [41, 42], suggesting the potential role of cytokines in chordoma progression. We will explore the levels and prognostic values of these cytokines in skull base chordoma, and their association with platelet associated indexes in the future study.

Some limitations exist in the current study. Considering the character of a single-centre retrospective study, additional large-scale, multicentre prospective studies are needed to verify our results and whether platelet is a potential therapeutic target for chordoma. In addition, the current study lacks mechanism studies explaining how these indexes affect the clinical outcomes of chordoma patients. Finally, the prognostic roles of other platelet indices and postoperative platelet-associated indictors, such as P-selectin [43] and postoperative PDW [44] in skull base chordoma were not analysed.

## Conclusions

Our data reveal that high levels of MPV and PDW are associated with poor OS in skull base chordoma patients. Importantly, PDW could independently predict patient outcomes, suggesting that PDW may act as a useful prognostic biomarker. In addition, our findings reveal the potential value of platelet-associated therapy in skull base chordoma.

## Supplementary information

**Additional file 1.** X-tile software was used to identify the optimal cut-off values of PLT, MPV and PDW for OS analysis in skull base chordoma. (a) The optimal cut-off value of PLT was 266. (b) The optimal cut-off value of MPV was 11.9. (c) The optimal cut-off value of PDW was 14.5. PLT, platelet count; MPV, mean platelet volume; PDW, platelet distribution width; OS, overall survival.

## Data Availability

All data used and/or analysed during the current study are available from the corresponding author on reasonable request.

## Abbreviations

PLT: Platelet count
MPV: Mean platelet volume
PDW: Platelet distribution width
OS: Overall survival
PDGF: Platelet-derived growth factor
HR: Hazard ratio
CI: Confidence interval
