# Supplementary figures and images for: Mean platelet volume and platelet distribution width serve as prognostic biomarkers in skull base chordoma: a retrospective study
Source: BMC Cancer. 2020 Oct 12;20:988. doi: 10.1186/s12885-020-07497-7 (PMC7552483; doi:10.1186/s12885-020-07497-7)

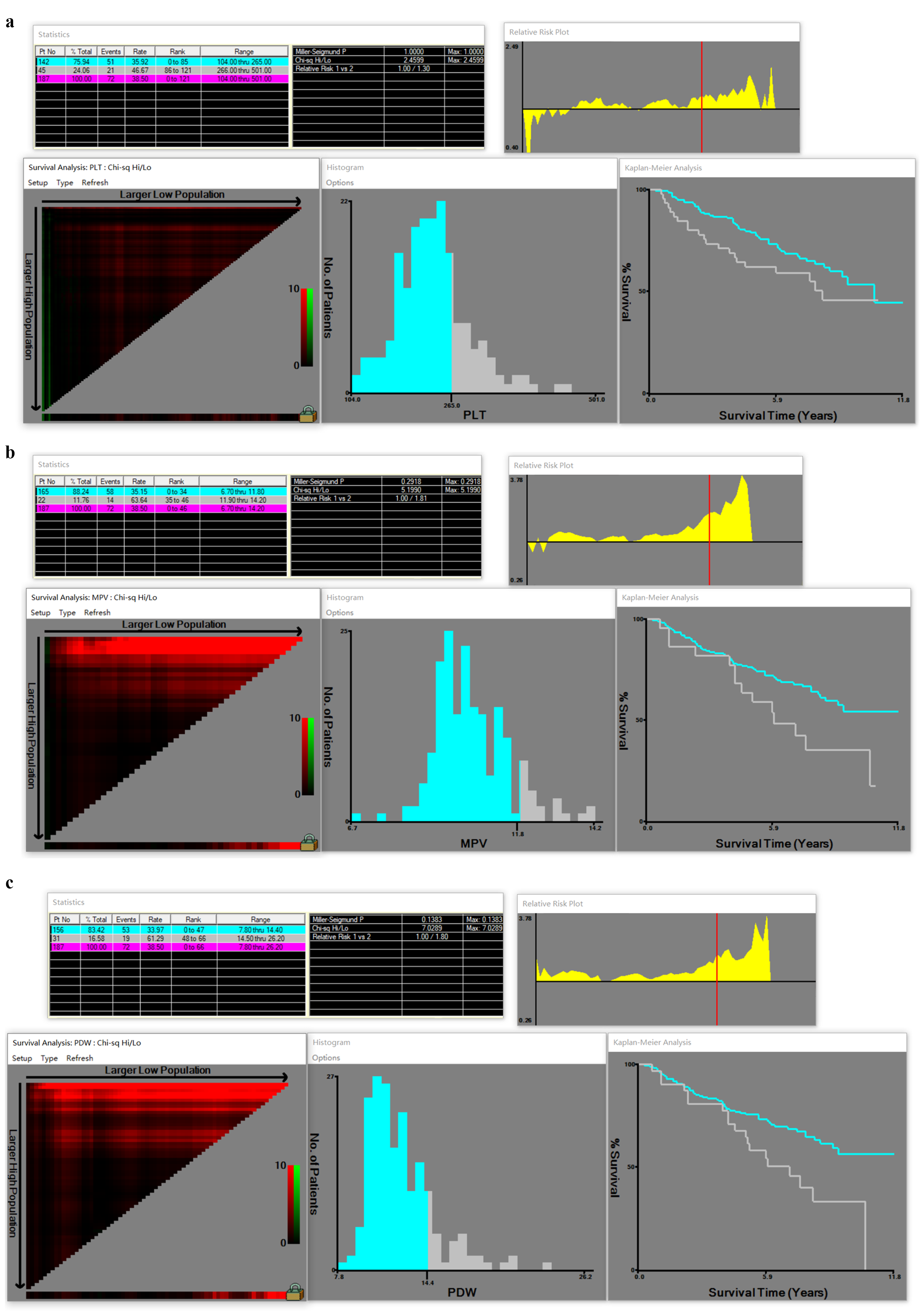

Supplement: Supplementary file 1 — Additional file 1. X-tile software was used to identify the optimal cut-off values of PLT, MPV and PDW for OS analysis in skull base chordoma. (a) The optimal cut-off value of PLT was 266. (b) The optimal cut-off value of MPV was 11.9. (c) The optimal cut-off value of PDW was 14.5. PLT, platelet count; MPV, mean platelet volume; PDW, platelet distribution width; OS, overall survival. [file 12885_2020_7497_MOESM1_ESM.tif]
